# Building school-based cardiovascular health promotion capacity in youth: a mixed methods study

**DOI:** 10.1186/s12889-015-1759-5

**Published:** 2015-04-25

**Authors:** Roberta L Woodgate, Corey M Sigurdson

**Affiliations:** Professor & Canadian Institutes of Health Research Applied Chair in Reproductive, Child and Youth Health Services and Policy Research, Faculty of Health Sciences, College of Nursing, University of Manitoba, Winnipeg, MB R3T 2N2 Canada; Ph.D. Candidate, Faculty of Graduate Studies, Applied Health Sciences, University of Manitoba, Winnipeg, MB Canada

**Keywords:** Youth, Mixed methods research, Positive youth development, Nursing, Health promotion, School program, Peer-led, Process evaluation

## Abstract

**Background:**

Essential to building cardiovascular health promotion capacity in youth, which extends into adulthood, are approaches that seek to empower, educate, and support. The Five Cs model of positive youth development (PYD) guided this study. This model represents the ability of youth to develop competence, confidence, connection, character, and caring when given the appropriate resources. The purpose of this two-year school-based feasibility study was to determine if providing a research intervention in the form of education, empowerment, and support build youth’s capacity for cardiovascular health promotion.

**Methods:**

A mixed methods case study design was used to evaluate the process, and outcome of a youth-led cardiovascular health promotion program. Twenty-six youth aged 12–13 years from a Canadian middle school took part in the study. Youth participating in this study planned, implemented, and monitored cardiovascular health promotion activities in four areas: smoking, physical inactivity, nutrition and obesity. Qualitative data was collected from the youth participants using three focus groups and individual reflective journals. Quantitative data was collected with the PYD.2, a self-report questionnaire that assesses positive youth development and consists of 5 subscales: character, competence, caring, connection, and confidence. The participants completed the PYD before and after the program to determine if there were any changes in PYD scores after the intervention. The quantitative data was analyzed using paired samples t-tests, and the qualitative data was analyzed using constant comparative analysis.

**Results:**

While the PYD scores showed no significant changes, the qualitative findings confirmed that the youth acquired increased awareness and understanding of cardiovascular health promotion initiatives. Four themes emerged from the qualitative data, (1) doing the right thing, (2) wanting to make a change, but feeling constrained, (3) I get it, and (4) The project has changed me! The intervention was found to be acceptable and feasible for the youth participants and their school.

**Conclusions:**

The contributions of this study were twofold. It generated evidence to support integrating positive youth development strategies into cardiovascular health promotion programs. Secondly, this study determined that the research intervention improved the participants’ knowledge and attitudes about cardiovascular health and were suitable for further implementation and testing.

## Background

Over the last decade, there has been a steady decline in deaths caused by cardiovascular disease in Canada [1]. However, heart disease and stroke remain two of the three leading causes of death [2], and 90% of Canadian adults continue to have one or more risk factors for developing cardiovascular disease [3]. Furthermore, there is increasing evidence that the development of cardiovascular disease risk is not limited to adulthood and begins with behaviors and experiences in childhood and adolescence [4,5]. For example, the Manitoba Youth-Health Survey reported that the youth are not practicing healthy behaviors [6]. Findings from this study showed: 4% of students (grades 9–12) eat the recommended daily servings of fruits and vegetables, and only 55% of male students and 41% of female students take part in the recommended amount of daily physical activity. Furthermore, this report found that 21% of male students and 21% of female students report being current smokers [6]. These unhealthy behaviors often extend into adulthood and contribute to long-term cardiovascular disease risk [4,5].

A primary goal of youth focused cardiovascular health promotion activities are to give age-appropriate information on the benefits of healthy behaviors including strategies addressing improving diet, eliminating tobacco exposure, and increasing physical activity [7]. There have been several studies published showing the effectiveness of school-based programs addressing these behaviors and preventing obesity [8-12]. However, limited data supporting the long-term effectiveness of these single behavior-based of programs is available [13]. Furthermore, risk factors for ill health across multiple domains are related [14-16] and best addressed through interventions targeting multiple domains [16,17]. Therefore, school-based cardiovascular health promotion programs need to incorporate programming that supports the development of multiple protective factors [14,18].

*Positive Youth Development* (PYD) is a strategy that can be used to connect cardiovascular health promotion with youth capacity for prosperity in school and the community [14]. The PYD approach to intervention planning and delivery can help youth develop critical analysis skills, positive sense of self and a sense of engagement in the community. The development of these skills can have a genuine impact on influencing positive social change [19-21]. Advocates of PYD maintain that youth with a positive sense of self are more engaged in their communities and are less likely to take part in risky behaviors [22,23], including behaviors that contribute to the development of cardiovascular disease.

PYD approaches to health promotion have been found to be useful in programming across a variety of domains [24,25]. Gavin et al. [26] conducted a systematic review of positive youth development programs that promote adolescent sexual and reproductive health. They found evidence of 15 programs that improved one or more sexual and reproductive health outcomes in adolescents. A randomized controlled trial of a PYD program aimed at preventing delinquency and drug use in Chinese adolescents found that program participants were less likely to engage in high-risk behaviors [27,28]. Dzewaltowski et al. [29] conducted a randomized controlled trial of the Healthy Youth Places intervention designed to promote increased physical activity and fruit and vegetable consumption in American middle schools. This intervention was guided by social cognitive theory and examined the development of personal and proxy agency [30] in youth as a strategy to build a healthy school environment. Youth assumed leadership roles in the intervention. This structure facilitated collaboration or proxy agency with adult leaders for implementing environmental changes the youth were not able to make on their own. Physical activity significantly increased in schools in the intervention group [29].

This paper describes a mixed methods study conducted to determine if providing a research intervention in the form of education, empowerment, and support build youth’s capacity for cardiovascular health promotion. Specifically, the study addressed the following research questions:

1. How do youth describe their experience participating in the research intervention?
2. How does participating in the research intervention change these youth’s lives?
3. Is there a difference in positive youth development after the research intervention?

The study applied the same premise described by Dzewaltowski et al. [29] specific to providing youth with environmental changes skills and personal efficacy as a health promotion strategy. Unlike *peer to peer* or *youth involved* health promotion programs where health professionals or other adult leaders are given primary responsibility for the conceptualization and development of the interventions [31-34], our study involved youth playing central roles in decision-making throughout all facets of health promotion programming. The participants identified, initiated, led, and monitored activities that addressed heart health promotion in four areas: smoking, physical inactivity, nutrition and obesity.

### Conceptual framework

The Five Cs model of PYD guided this study. The 5 Cs (i.e., confidence, competence, character, connection, and caring) is the most empirically supported model of PYD [35] and emphasizes that all youth have strengths and when provided with resources, these strengths are enhanced [36]. Bower’s et al. [37] definitions of the 5 Cs are presented in Table 1. The 5 Cs model acknowledges that developing healthy behaviors and beliefs is contingent on a reciprocal relationship between a developing young person and the areas of his or her life that encourage and promote healthy development [36]. A PYD approach builds youth capacity by fostering the development of skills through learning partnerships and action to create change to promote lasting effects. Cargo et al. [19] point out that capacity in youth can be enhanced by youth taking part in programs that are both challenging and supportive. This model supports the study premise that providing youth with education, empowerment, and support, increases youth capacity for cardiovascular health promotion.

**Table 1:** **The five Cs of positive youth development** [32]

**Characteristic**	**Definition**
**Competence**	Positive view of one’s actions in domain specific areas including social, academic, cognitive, and vocational. Social competence pertains to interpersonal skills (e.g., conflict resolution). Cognitive competence pertains to cognitive abilities (e.g., decision-making). School grades, attendance, and test scores are part of academic competence. Vocational competence involves work habits and career choice explorations, including entrepreneurship.
**Confidence**	An internal sense of overall positive self-worth and self-efficacy; one’s global self-regard, as opposed to domain specific beliefs.
**Connection**	Positive bonds with people and institutions that are reflected in bidirectional exchanges between the individual and peers, family, school, and community in which both parties contribute to the relationship.
**Character**	Respect for societal and cultural rules, possession of standards for correct behaviors, a sense of right and wrong (morality), and integrity.
**Caring**	A sense of sympathy and empathy for others.

Derived from Lerner et al. [24] and Roth et al. [72].

## Methods

This study was approached using an embedded mixed methods case study design. Researchers use case study designs when examining a bounded phenomenon intending to provide thick description that is meaningful and applicable to practice and future research [38-42]. Besides understanding a particular situation in great depth, case studies are also valuable when the evaluation strives to capture individual differences or unique variations from one setting or situation to another [41,42]. For the purpose of this study, the case unit (i.e., building youth capacity for cardiovascular health promotion through a research intervention), was examined in one school. The quantitative data were embedded within the larger qualitative case study. Qualitative data explored how the research intervention was experienced by the participants. The quantitative data was embedded to determine if the research intervention influenced positive youth development in the participants.

### Participants and recruitment

The primary participants were youth recruited from one classroom in a middle school (junior high) in a medium-sized urban center. The first author previously conducted a research project in the same school district and had established rapport and a trusting relationship with school administration. Youth enrolled in that school district had diverse cultural and socioeconomic backgrounds. Inclusion criteria included youth who: (1) were between 11–15 years of age (2) had verbal and written consent provided by their legal guardian, (3) spoke English, and (4) gave their assent. Interested youth were asked to email or telephone the research coordinator who further explained the study and obtained parental consent and youth assent. The research coordinator started recruitment at the beginning of the school year.

### The HEART intervention

**Figure 1:**
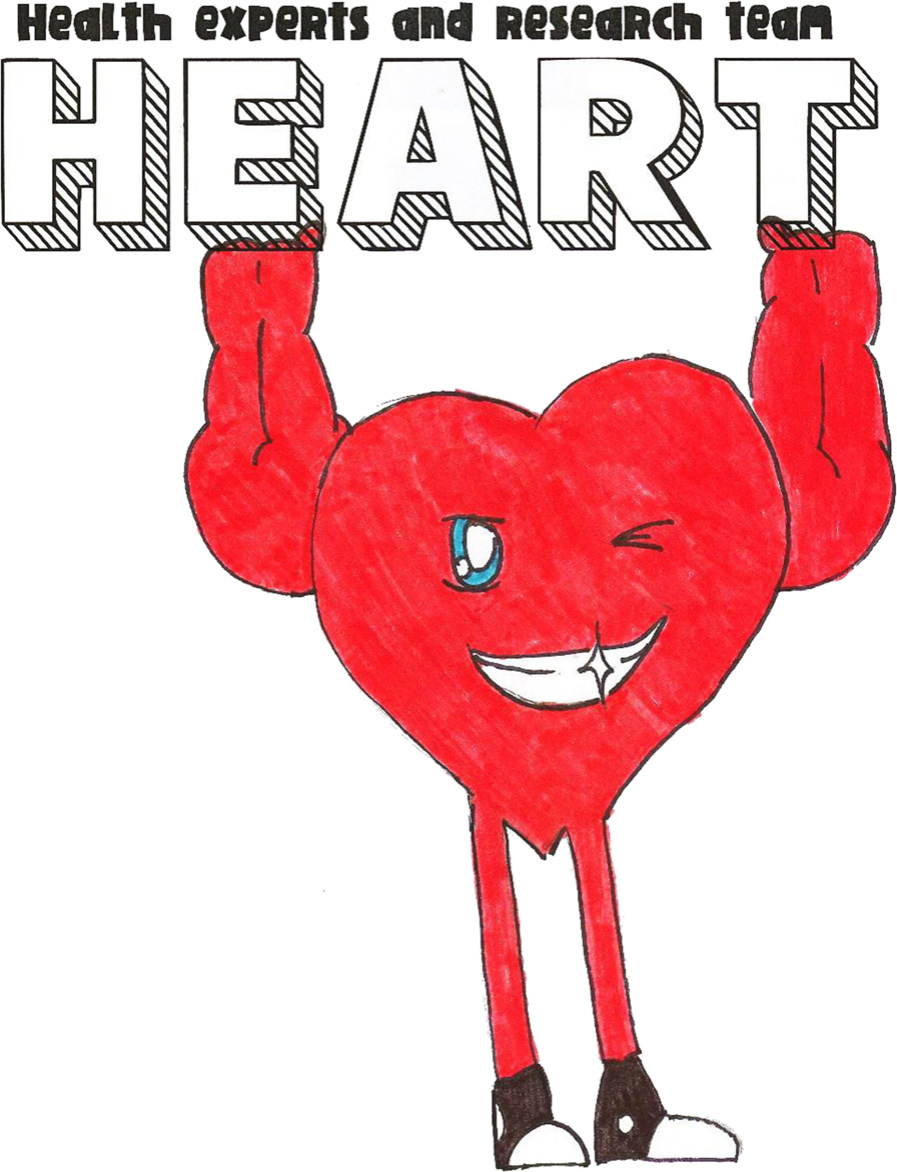
Health experts and research team (HEART) logo.

The research intervention, as education, empowerment, and support was structured to build youth’s knowledge and skills for cardiovascular health promotion and to enable them to identify, initiate, lead, and monitor cardiovascular health promotion activities. The study spanned two school years (22 months). The participants were responsible for identifying, initiating, leading, and monitoring activities that addressed health promotion in four areas: smoking, physical inactivity, nutrition, and obesity. The students were tasked with creating a group identity, and they chose the name *Health Experts and Research Team* (HEART) and created a logo pictured in Figure 1.

In order to develop group and individual capacity, the youth were provided with training that focused on developing leadership and team building skills, cardiovascular health knowledge, health promotion strategies (e.g., social marketing, advocacy as a means to effect change), and action plans (e.g., evaluation strategies). The training was meant to both educate and empower youth so that they were able to develop, undertake, and evaluate multi-factorial cardiovascular health promotion activities while developing personal skill. Two teachers from the participating school took part in the study. The teachers developed and integrated curriculum unique to the research process, social marketing, and heart health promotion into the students’ lessons. The teachers sought out advice from researchers to develop the curriculum. The teachers also assisted the youth participants in implementing their activities and functioned as the primary staff contact at the school.

In addition to the existing teacher delivered health and physical education curriculum, students were provided with six intervention activities as described in Table 2. These activities included a heart health research workshop led by the research team that involved presentations related to conducting research as well an exercise that provided students with experience in conducting a research study. The goal of this exercise was impart knowledge to foster positive interaction between the students and the research team.

**Table 2:** **Intervention activities**

**Activity**	**Description**
**Heart Health Research Workshop**	This workshop served as the official commencement of the study. The workshop included a number of presentations related to conducting research as well as *hands-on* experience for students in conducting a research study.
**Art Gallery Workshop**	The gallery uses art as a tool for community, social, economic and individual growth. One of its aims is the promotion of youth art as its own genre. The purpose of this workshop was to increase students’ knowledge and skills in constructing effective heart health promotion messaging.
**Healthy Food Workshop**	This workshop was held in a multi-faceted facility that provides accurate and professionally led classes on food-related topics. The purpose of this workshop was to increase students’ understanding of healthy heart food preparation. The students learned healthy meal choices and participated in preparing healthy dinners that they were able to take home and share with their families.
**Heart Gets Fit Workshop**	The purpose of this workshop was to increase the students’ understanding of benefits of physical activity. The students participated in a fitness class and visited the university athletic facilities and kinesiology lab.
**University Research Day**	The purpose of this day was to introduce the participants to applied health research. Participants visited a food science facility and had hands on experience in a health sciences laboratory.
**Web-Based Health Promotion Project**	Students worked in groups of three. Each group was assigned a simulated middle school client with sub-optimal health. The students were tasked with creating a fitness assessment, a nutrition plan and a physical activity plan, with each student responsible for one aspect of the plan. The purpose was to create a strategy to promote their client’s health. The students were provided with current, evidenced based web resources in order to complete their projects.

### Data collection

Multiple data collection methods that included both quantitative and qualitative measures were used to evaluate both the processes and the outcomes of the intervention. The principle investigator (RW) and the research coordinator were responsible for collecting the data.

### Focus groups

The cohort took part in three focus group interviews. Focus groups are useful for studies of complex issues as they give an opportunity for instances of interchange between contrasting perspectives [43-47]. Using focus groups allows individuals to build on other’s comments, a characteristic that was desirable for this situation. The first focus group was conducted before introducing the intervention, the second midway through the intervention, and the third after the intervention was completed. Conducting three focus groups interviews afforded the opportunity to arrive at a detailed understanding of building youth cardiovascular health promotion using a PYD approach.

Interviews guides were developed to yield both process and outcome data. The focus of all three interviews was to ascertain youth’s perspectives on the processes involved in identifying, initiating, leading, and monitoring heart health promotion activities. As well, for the second and third interviews, questions specific to activities implemented, conditions impacting youth’s activities, challenges encountered, what worked and what did not work, and the impact that the intervention had on youth and school, were also asked. Separate focus group interview guides were developed for each data collection time point. Acknowledging that a six to 12 member focus group is considered the most appropriate size in facilitating the exchange of ideas [43-46], the research team conducted three interviews at each of the three data collection points. The groups lasted 90–120 minutes, digitally recorded, and transcribed verbatim to preserve their authenticity.

### Journals

The youth participants completed written journals following each of the intervention activities. Journaling is useful for exploring the ongoing experiences of participants [48].

### Demographic form

The youth participants were asked to complete a demographic form to obtain a profile of the study’s sample.

### Positive Youth Development (PYD) measure

The PYD.2 [22,23,36,37,49] was the primary outcome measure. The PYD.2 measure is a self-report questionnaire that assesses positive youth development in children in grades five to seven. It contains 83 items comprising of five subscales: character, competence, caring, connection and confidence [22,23,36,37,49]. Work to date establishes this tool as a valid measure of PYD across early and older adolescence with both convergent and discriminant validity and high levels of internal consistency (alpha > 0.70). The participants completed the PYD measure before and after the intervention to determine the impact that the intervention had on promoting positive development in youth.

### Ethical considerations

The research team maintained ethical standards throughout the study. Attention to issues of parental informed consent and youth assent, confidentiality, and potential vulnerability, and sensitivity of youth participants were carefully considered. Written consent was secured from all parents and teachers, and assent from youth before beginning data collection. All participants were identified by code numbers to protect their identity. Youth were provided with instructions of the “do’s and don’ts” of acceptable behavior for interacting on a project team. Each participant received an honorarium in appreciation of their time spent on the study. The authors received ethical approval from the Education/Nursing Research Ethics Board at the University of Manitoba.

### Data analysis

#### Qualitative data analysis

The qualitative data that emerged from the journals and focus group interviews was imported into NVivio [50] to facilitate organization and coding. In keeping with the qualitative tradition, the research team analyzed the data as it was collected. The qualitative data was analyzed by both authors to facilitate arriving at a comprehensive understanding while the first author assumed the overall responsibility for the data analysis. The constant comparative method of data analysis was used to develop inductively derived themes and categories. In the constant comparative method all data is coded or given meaning, codes are then revised and/or the data is re-coded. Finally, the codes are aggregated and clustered into themes and categories [51-54]. The goal was to identify and develop themes that illuminated the processes and outcomes associated with whether youth taking part in the study experienced increased capacity for cardiovascular health promotion and if the youth experienced increased cardiovascular health promotion awareness and actions. Meetings were held to discuss emerging codes and themes with the goal of both investigators arriving at consensus on the final themes.

#### Quantitative data analysis

Quantitative data analysis was performed with SPSS [55]. Basic descriptive statistics, including means and percentages were calculated for the demographic data and paired-samples t-tests were used to decide if there were any statistically significant changes in any of the 5C scores following the intervention.

#### Rigor

The research team undertook several measures to enhance the rigor of this study. These measures included prolonged engagement in the phenomenon under study, multiple data collection methods, attention to potential biases, and iterative analysis [56].

## Results

The sample comprised 26 participants. All participants took part in the focus groups, and 20 completed the PYD.2 (six students declined completing the PYD.2). The number of male participants was 14 (53.8%), and the number of female participants was 12 (46.2%). At the outset of the study, 24 of the participants were age 12 (92.3%), and 2 (7.7%) of them were age 13. The majority of the participants (88%) were living with both parents, and 72% reported that they had at least a fair amount of heart health knowledge. The details of this information presented in Table 3.

**Table 3:** **Demographic profile of participants (N = 26)**

**Characteristic**	**N**	**Percentage**
**Age**		
12 years old	24	92.3
13 years old	2	7.7
**Sex**		
Male	14	53.8
Female	12	46.2
**Ethnicity**		
European	9	37.5
Canadian Aboriginal	4	16.7
Other (Asian, African, Arabic, Canadian)	11	45.8
**Residential Situation**		
Single-parent household	1	4.0
Two parent household	22	88.0
Other (e.g. living with grandparents, stepparents)	2	8.0
**Knowledge about heart health**		
A little	5	20.0
A fair amount	18	72.0
A lot	2	8.0

### Pre-intervention focus groups

Two themes emerged from the pre-intervention focus groups: (1) *doing the right thing* and (2) *wanting to make a change, but feeling constrained*.

### Doing the right thing

Evidence from the pre-intervention focus group revealed that the participants viewed cardiovascular health as a process of individual participation in healthy activities. As was reported in earlier work by Woodgate [57], youth spoke in terms of “dos” and “don’ts”, and “doing the right thing” for promoting cardiovascular health. The conversation was dominated by discussion of personal lifestyle practices related to choosing healthy food and participating in regular exercise. The participants knew of the consequences associated with unhealthy lifestyle choices had on their hearts and felt personal responsibility for achieving and maintain cardiovascular health. One participant stated,

*Yeah, I think like most of the time like we are responsible for our own health and like if we, like say we eat something really fatty then you’re putting that in your body and it’s obviously going to give you excess sugars. If you don’t work it off and you’re just eating all this stuff and you’re just going to go sit there. And it just sits there and it’s eventually going to turn to fat and it kind of goes straight to your veins and arteries. You’ve got to work it off after or…get a heart attack.*

All youth also revealed feelings of frustration and sadness when they saw friends and family members demonstrating unhealthy behaviors. The feeling of frustration was evident when it came to others smoking cigarettes. One youth stated,

*I don’t know cause I know some people in my family that do smoke and the anti-smoking commercial came on and I just gave them this look and they didn’t look at the TV, they just didn’t, didn’t want to look at it. Like honestly if my dad saw that commercial he’d probably just think, “oh that, that's probably never going to happen to me”, something like that…Smoking is not good for you, like every smoke you have, wastes five minutes of your life* (tears in eyes)

Another commented,

*During review and rate (specific to anti-tobacco smoking ads), I felt really sad when I saw all of the ads on smoking! Ad number 2, 6, 8, 11 and 12 really touched my heart to see a man who has oral cancer and lost his tongue, to see all those people suffering every minute of their life due to smoking, to see children crying because they don’t want their mothers and fathers to smoke!*

### Wanting to make a change, but feeling constrained

The pre-intervention focus groups also revealed that while youth wanted or desired to lead healthier lifestyles, they also felt restricted in doing so due to barriers in their daily lives. Most often the barriers were associated with their home life, and their parents’ inability to provide a healthy environment. A common topic amongst youth was a wish to have more food they defined as “healthy” in their homes including more fruit and vegetables and less meat and “junk” food. One participant expressed,

*I did tell my mom “okay buy more vegetables, cause I want to eat all my vegetables”…but we eat more meat…Well like, like the pork chops and everything, we have rice and vegetables, but there’s not that much of vegetables but more meat. There’s like more than one serving, it’s like three servings of meat.*

Some youth also noted that due to financial constraints, they were unable to take part in organized sports or join athletic facilities (e.g., private gyms),

*My family can’t afford anything like that (i.e., going to the gym)…I wish we could. I wish the gyms were cheaper.*

Youth identified second-hand smoke because of parents smoking in their homes as a barrier to a healthy lifestyle not only for themselves but also for their family members,

*Whenever I go to my dad’s house, I inhale smoke, and not just me, my cousins, my grandparents and my friends…*

A common wish among youth who had parents who smoked was for their parents to stop smoking,

*My wish for my house to be healthy is for my mom and dad to stop smoking. But then, but then once they get on it and they try and quit that makes them more miserable. Yeah and then they just smoke more.*

Youth also identified barriers associated with their school environment including nearby fast-food restaurants, a small gym, and students who smoke around the school. In particular, the school’s cafeteria was seen as a barrier to a healthy lifestyle. Comments such as the following were frequent,

*Every Thursday is pizza day and some kids like have six pizzas, five pizzas. Yeah six pieces of pizza, it’s just almost like…I like more vegetables on the menu…All they have really is like rice and vegetables, but it’s only like rice, like that’s pretty much all you see in it.*

### Post-intervention focus groups & journal entries

Two themes emerged from post-intervention focus groups and journal entries: (1) *I get it,* and (2) *The project has changed me!*

### I get it

Analysis of the post-intervention focus groups and journal entries revealed that the youth participants were engaged and enthusiastic about the research intervention. One participant stated,

*The concept of research has changed for me. I thought the professors would do their research physically on us. Like ask questions, watch we do in school etc. But instead, we were doing our own research, and we showed them our results and they record it.*

Furthermore, participants were found to have a basic understanding of the broader determinants of health. Another participant stated,

*I talked about how my family was meat lovers! Dr. Roberta was asking my classmates and I questions about what the environment looks like and what would you change the environment! I enjoyed talking to Dr. Roberta, talking about how the environment looks to me and my classmates!*

### The project has changed me!

Analysis of the post-intervention focus groups of journal entries also showed that the youth participants perceived personal benefits from participating in the intervention. One youth participant stated that,

*I think HEART has affected my life quite a bit. I think about the foods I eat and how I want to live a healthier lifestyle. The research has affected my opinions about heart health. I know that choosing to smoke will give me cancer and/or brain damage. I thought the overall value of this project was to get kids thinking more about a subject that isn’t French, gym, etc. It gets them thinking how their lifestyle is and helps them choose how they will live/plan their lifestyles.*

Participants found that taking part in the research intervention increased their ability to promote the cardiovascular health of others as revealed in the following comments,

*Now I can help my peers and my dad to stop smoking, I can now tell what can happen if they still smoke.*

*I was so sad seeing people suffer from smoking and that the ad I picked represented suffering. Every minute, it was so sad. It was about this guy sitting at a table having an oxygen tank and he was coughing so hard and it was so sad seeing that…I will tell my friend to never smoke and I will never smoke. I learned that to never smoke because it will have a big effect to everyone and me.*

*Now I can cook healthy pizzas or chicken parmesan, or I can cook for my peers and promote heart health at the same time by telling them what is healthy to eat, or what is not healthy to eat.*

Overall youth revealed that benefits from participating extended beyond heart health promotion as reinforced by the following two comments,

*I think this project is valuable in many ways and it is definitely worth learning for future references. Especially at our ages as it expands our minds to new levels!*

*I feel this project gave the students a chance to act mature, feel like an adult and try new things. So the overall educational value is very high!!!*

### Pre-intervention to post-intervention effects

Each of the 5 Cs measures was converted to a score on a 100-point scale with a larger number reflecting possessing a greater level of the construct. Scores of 75 and above were considered indicative of the construct being present for this study. The results show that 17.4% (n = 4) of the youth participants already possessed all five Cs before the intervention. Sixteen did not possess all five Cs, but three possessed four Cs, seven possessed three Cs; one possessed two Cs, four possessed one C, and one participant did not indicate possessing any of the five characteristics at all.

Mean scores were calculated for each of the five constructs. Paired-samples t-tests were used to decide if there were any statistically significant changes in any of the 5C mean scores following the intervention and the results are reported in Table 4. Participants with missing data were excluded from the analysis, complete pre and post-test data was available for a total of 20 participants. Significance was set at p < 0.05 and data are reported as mean ± standard deviation. There were no statistically significant changes in any of the 5C scores following the intervention. There were decreases in the character, competence, connection, and confidence scores and an increase in the caring score.

**Table 4:** **Paired** ***t-*** **test results for post-intervention change (N = 20)**

**PYD Measure**	**Before**	**After**	**Change**	**95% CI**	***t (p)***
	**Mean (SD)**	**Mean (SD)**			
**Caring**	69.99 (16.67)	71.49 (18.55)	1.51	−7.71, 10.73	0.34 (0.74)
**Character**	77.30 (15.96)	75.33 (9.70 )	- 1.97	−9.75, 5.81	−0.53 (0.60)
**Competence**	69.73 (16.24)	69.27 (9.87)	- 0.46	- 6.79, 5.86	−0.15 (0.88)
**Connection**	78.24 (11.00)	77.67 (9.94)	- 0.57	−6.44, 5.31	−0.20 (0.84)
**Confidence**	82.58 (12.87)	74.69 (12.79)	−7.89	−16.09, 0.30	−2.02 (0.06)

### Process evaluation

The duration of the program appeared to be a barrier to participation. School-based health promotion interventions have previously been successful when offered over ten-month periods [33,58]. The original intent of the project was to provide a ten-month (one school year) intervention program consecutively to two separate cohorts. However, the school involved in the project requested that one class take part in the intervention over two consecutive ten-month school years. In keeping with the participatory nature of this project, the research team complied with the school’s request and delivered the intervention and collected data from the same class over two years. This was possible as the participants remained as a group as they progress to the next grade.

Overall the staff and students were less motivated in the second year of the project. When asked about participation in year two, it became apparent that the participants would have preferred a higher intensity program of shorter duration. As noted by the following comments,

*Well the breaks that we took weren’t really helpful.*

*I like everything about the project. It is just that this year we did less things than last year.*

*I don’t have any negatives towards HEART. The only one is that I really wish we would have done more!*

The perception of increased workload by teachers was found to be a barrier to implementation in a previous school-based study [59], and the decision to offer the intervention activities during school hours was made at the request of the teaching staff. The intervention was provided during school hours and all students present in the class routinely participated. Offering the intervention during school hours is believed to have been a major contributing factor to the high participation rates. The integration of health promotion programming into the existing school schedule has been found to facilitate program involvement in similar studies [60,61].

## Discussion

By applying used a mixed methods participatory design to evaluate a PYD approach to cardiovascular health promotion, the contributions of this study were twofold. First, it generated evidence to support integrating positive youth development strategies into cardiovascular health promotion programs. Secondly, this study determined that the research intervention improved the participants’ knowledge and attitudes about cardiovascular health and were suitable for further implementation and testing.

The approach presented combines youth cardiovascular health promotion with activities to build the participant's capacities for increased competence, confidence, connection, character, and caring. The presence of these traits is known to increase youth prosperity in school and the community [14], and most importantly reduce participation in risky behaviors across multiple domains [62,63]. Moreover, this approach provides youth with life skills that may protect them against the psychosocial risk factors for developing cardiovascular disease. The evaluation data has demonstrated the potential value and participant acceptability of youth cardiovascular health promotion programs delivered within a PYD framework.

Youth participants did not initially recognize that the project was centered on using and developing their innate abilities [21]. However, qualitative data collected during and after the intervention revealed that participants felt connected to the project and had developed positive relationships with research team members. Connectedness and positive adult relationships have been found to contribute to positive behavioral and psychosocial outcomes in other school-based studies [29,64]. We believe this sense of connectedness contributed to the participants’ acceptability of the project.

The youth participants were actively involved in leading the intervention. This decision to develop a youth-led intervention was made in recognition of evidence that youth inherently possess valuable knowledge and skills and want to contribute to meaningful projects [65,66]. Youth-led health promotion engages youth in social advocacy and community development while enhancing feelings of control and ownership over projects [31,67]. Youth-led programs offer participants the opportunity to become involved in program development and implementation. Engaging youth in a meaningful way increases the likelihood that activities are acceptable to youth [68]. Furthermore placing youth in leadership roles has been found to enhance their ability to affect environmental change [29].

There is also evidence suggests that youth-led health promotion programs are more effective than adult-led programs [69]. Youth-led health promotion programs also take advantage of the inherent close communication and strong peer influence that occurs during childhood and adolescence [34]. Both the generation and uptake of significant knowledge is critical to changing practice and sustaining cardiovascular health promotion awareness and actions [70].

The participants entered the study with basic knowledge of the major modifiable risk factors for poor cardiovascular health. However, similar to Woodgate’s [71] study of youth’s knowledge of cancer risk, participants were less aware of non-modifiable risk factors such as genetics and environmental factors. Consistent with the findings of Woodgate [57], the participants in this study were not fully aware of the broader social determinants of health. This focus on lifestyle choices and personal responsibility has the potential to lead to adverse consequences for youth, such as feelings of frustration, guilt or blame. Particularly in those who cannot make individual lifestyle choices due to personal circumstance [57]. Feelings of frustration were evident when participants discussed witnessing others participating in unhealthy behaviors. Participants could identify unhealthy behaviors in others, but they could not affect any change. Having the knowledge of the adverse effects of certain behaviors is positive however youth can be negatively affected when witnessing unhealthy behaviors in others, especially friends and family members. Woodgate [72] previously found that young people who were routinely exposed to tobacco smoke are negatively affected by fear for their health and the health of the smoker. As this study progressed youth developed an increased awareness and understanding of heart health promotion initiatives and strategies in the areas of smoking, physical inactivity, and obesity. Youth developed an increased awareness and understanding of social marketing and advocacy skills as a means to effect change specific to heart health promotion.

The pre-intervention to post-intervention testing did not reveal any significant changes in the participants’ PYD scores, but this group already possessed high levels of PYD characteristics before the intervention. These high pre-test findings may be reflective of the positive and supportive environment of the school. In fact, the willingness of school administration to take part in this research project may indicate that this school is atypical. The fact that there were no significant decreases in PYD scores after the intervention may be noteworthy as a longitudinal study of PYD found that scores tend to decrease slightly during adolescence [36]. A decrease was not seen in this study. Furthermore, the qualitative themes that emerged from the study are indicative of increased PYD.

### Strengths and limitations of the study

There are several limitations to this study including the small sample size and the lack of experimental design. Furthermore, the intervention was delivered to a group that was not deemed to be at high risk. Tailoring intervention programs to meet the needs of high-risk individuals has been found to increase program success [73].

Using mixed methods helped to achieve a broader perspective of building youth’s capacity for cardiovascular health promotion and yielded details about study processes and outcomes that neither method can achieve alone [74,75]. A case study design afforded the opportunity for an in-depth evaluation of the case on both process and outcome. Despite the limitations of this study, we have presented mixed methods study of a PYD approach to adolescent cardiovascular health promotion where the intervention moves beyond the *absence of bad behaviors* to include identifying and enhancing youth’s assets and potential. Future research is warranted using a larger sample size to evaluate the effectiveness of the intervention on a larger scale [24]. A larger sample size would also allow for analysis of subgroups including high-risk, gender, and socioeconomic status [76].

## Conclusion

Developing and testing interventions that can build youth’s capacity for cardiovascular health promotion are critical to reducing the burden of disease for future generations in Canada. School-based focused health promotion programs are more likely to succeed when youth are involved in all stages including planning, implementation, and evaluation. Findings from this study may be used to inform future decisions about using a PYD approach to adolescent cardiovascular health promotion. A PYD approach to youth cardiovascular health promotion not only provides youth with knowledge of cardiovascular health, it fosters the development of life skills such as leadership and advocacy that support long-term health. Youth are concerned about personal health, the health of those around them and can make meaningful contributions. As one participant from the study stated,

*I think kids make good researchers because they have yet to learn. As they grow, they are curious about the surroundings they are in. They want to know by doing that; they do their own research with the help of others. We have a brain, we might as well use it!*
